# Downregulation of miR-29c promotes muscle wasting by modulating the activity of leukemia inhibitory factor in lung cancer cachexia

**DOI:** 10.1186/s12935-021-02332-w

**Published:** 2021-11-27

**Authors:** Kairu Xie, Hairong Xiong, Wen Xiao, Zhiyong Xiong, Wenjun Hu, Jiaxin Ye, Ning Xu, Jian Shi, Changfei Yuan, Zhixian Chen, Daojia Miao, Xiaoping Zhang, Hongmei Yang

**Affiliations:** 1 Department of Pathogenic Biology, School of Basic Medicine, Tongji Medical College, Huazhong University of Science and Technology, Wuhan, 430030 Hubei Province China; 2 Wuhan National Laboratory for Optoelectronics, Huazhong University of Science and Technology, Wuhan, China; 3 Department of Urology, Union Hospital, Tongji Medical College, Huazhong University of Science and Technology, Wuhan, China; 4 Department of Clinical Laboratory, First Affiliated Hospital of Anhui Medical University, Hefei, China

**Keywords:** Cachexia, Muscular atrophy, miR-29c, Leukemia inhibitory factor

## Abstract

**Background:**

Cancer cachexia is a wasting disorder characterized by significant weight loss, and is attributed to skeletal muscle weakness. In the process of cancer development, microRNAs act as oncogenes or tumor suppressors. Moreover, they are implicated in muscle development and wasting. This study sought to explore the mechanisms and correlation between miR-29c and muscle wasting in lung cancer cachexia.

**Methods:**

Data for expression analysis were retrieved from the Cancer Genome Atlas (TCGA) database. qRT-PCR analyses were performed to explore the expression levels of miR-29c and Leukemia Inhibitory Factor (LIF). Lewis lung carcinoma (LLC) cell line was used to establish a cachexia model to explore the functions of miR-29c and LIF in lung cancer cachexia. Furthermore, in vitro (in C2C12 myotubes) and in vivo (in LLC tumor-bearing mice) experiments were performed to explore the mechanisms of miR-29c and LIF in lung cachexia.

**Results:**

Analysis of the lung cancer cachexia model showed that miR-29c was down-regulated, and its expression was negatively correlated with muscle catabolic activity. Overexpression of miR-29c mitigated the cachectic phenotype. Mechanistic studies showed that LIF was a direct target gene of miR-29c, and LIF was upregulated in vitro and in vivo. Analysis showed that LIF promoted muscle wasting through the JAK/STAT and MAP-kinase pathways.

**Conclusions:**

The findings indicated that miR-29c was negatively correlated with the cachectic phenotype, and the miR-29c-LIF axis is a potential therapeutic target for cancer cachexia.

**Supplementary Information:**

The online version contains supplementary material available at 10.1186/s12935-021-02332-w.

## Background

Cancer cachexia is a wasting syndrome characterized by irreversible weight loss and frailty that affects approximately half of cancer patients. This syndrome results in progressive functional impairment and accounts for approximately 20% of all cancer mortalities [1]. Notably, the syndrome cannot be fully reversed by conventional nutritional support [2]. Therefore, identifying crucial molecules implicated in the pathogenesis of cancer cachexia may aid in the development of a therapeutic strategy to alleviate or prevent cachexia.

Cachexia is mainly associated with skeletal muscle weakness, characterized by progressive body weight loss, reduced muscle fiber cross-sectional area (CSA), and dysregulated protein balance [1, 2]. Recent findings suggest that muscle wasting in cachexia is likely to be the major cause of death in patients with cancer. Consequently, the preservation of muscle mass may increase survival [3, 4]. There are many metabolic alterations are responsible for the decrease of the muscle mass, such as increased energy expenditure and proteolysis, ensuing from activation of ubiquitin–proteasome pathway (UPP) and autophagy-lysosomal pathway (ALP) [5]. The variations of UPP and ALP are the main cancer cachexia hallmarks [6]. UPP effectively degrades proteins through ubiquitin, ubiquitin-conjugating enzyme or E1 enzyme, ubiquitin-activating enzyme or E2 enzyme, ubiquitin ligase or E3 enzyme, protease, and its substrate [7]. The two muscle-specific E3 ubiquitin ligases in skeletal muscles include muscle atrophy F-box protein (atrogin1/Fbxo32) and muscle RING finger containing protein1 (MuRF1/Trim63) [8]. Atrogin1 and MuRF1 are the key proteins in the development of muscle atrophy in cachexia [9, 10]. ALP uses autophagosomes to engulf ubiquitinated proteins and organelles then subsequently transfers them to autolysosomes, where the proteins are enzymatically degraded [11]. During this process, autophagy markers (LC3 and P62) are activated in the cachectic muscles of tumor-bearing mice [5]. These findings imply that UPP and ALP are essential components of muscle catabolism activation during cancer cachexia. However, the mechanisms through which cancer activates muscle wasting have not been fully elucidated.

Chemotherapeutic strategies for inhibition of the cachectic phenotype are not effective owing to the various muscle catabolism mechanisms. Therefore, there is a need for identification of effective molecular targets of cachexia [12, 13]. miRNAs are short noncoding RNAs that act as oncogenes or tumor suppressors by modulating gene transcription or post-transcriptional processes [14, 15]. miRNAs regulate muscle metabolism, growth, and regeneration in cellular and animal models of muscle wasting during cancer cachexia [7]. miRNAs such as miR-21 and miR-206 promote or inhibit skeletal muscle atrophy by playing key roles in muscle development and skeletal muscle wasting in cachexia [16]. Studies report that members of the miR-29 family act as a potent tumor suppressor gene in various cancer types [17], including lung cancer, pancreatic cancer and colon cancer [18–20]. In addition, NF-κB–YY1–miR-29 axis regulates skeletal myogenesis. miR-29 functions as an enhancer of differentiation during myogenesis in muscle cells [21]. Overexpress miR-29c in tibialis anterior increase muscle mass and improve muscle function by stimulating satellite cell proliferation and repressing atrophy‐related genes [22]. However, studies have not fully elucidated the role of miR-29c in cancer cachexia. We therefore hypothesize that repressed miR-29c may play a role in muscle wasting in cancer cachexia. We use Lewis lung carcinoma (LLC) cell line to establish cachexia model both in vitro and in vivo (classic cancer cachexia model [8, 11, 23]) to explore the functions of miR-29c and related signaling pathways.

## Methods

### Materials

The LLC and 293T cell lines were purchased from The American Type Culture Collection (ATCC, USA). Murine C2C12 myoblast cells were obtained from the Cell Bank/Stem Cell Bank, Chinese Academy of Sciences. Primers and oligonucleotides, including mmu-miR-29c-3p mimic, mmu-miR-29c-3p inhibitor, siRNA specific for LIF, and negative control were purchased from RiboBio, Guangzhou, China. Primary antibodies used for western blotting analysis include anti-Atrogin1 (Abcam, ab168372), anti-MuRF1 (proteintech, 55456-1-AP), anti-LC3 (proteintech, 14600-1-AP), anti-P62 (ABclonal, A19700), anti-GAPDH (Proteintech, 60004-1-Ig), anti-LIF (Abcam, ab113262), anti-p-p38 (Cell Signaling Technology, 4511T), anti-p38 (Cell Signaling Technology, 9212S), anti-p-STAT3 (ABclonal, AP0070), and anti-STAT3 (ABclonal, A1192). Primary antibodies used for Immunohistochemistry: anti-LIF (ABclonal, A1288). Primary antibodies used for fluorescence microscopy analysis: anti-MHC (R&D Systems, MAB4470).

### Animals

Ethical approval for use of animals in the current study was obtained from the Institutional Animal Care and Use Committee of Tongji Medical College, Huazhong University of Science & Technology. C57BL/6 male mice (6–10 week old [8]) were purchased from SPF Biotechnology Co., Ltd, Beijing for use in animal experiments. Mice were fed with a standard irradiated rodent chow diet at the same time every day and kept in 12 h light room at 22 °C, lights turn off during 6 a.m. to 6 p.m. Mice were randomly allocated into tumor-bearing (TB) and non-tumor bearing (control; CN) groups and were labeled with clear ear tags to avoid mistakes (no blinding). The average body weight for each group was similar. Lewis lung carcinoma (LLC) cells (5 × 10^6^ per mouse) were injected subcutaneously over the flank, whereas mice in the CN group were only administered with the vehicle (PBS) [8]. We monitored the food and water intake of mice every day, and measured the weights of mice every three days. Mice were euthanized with a 30% CO_2_ flow rate for 1.5 min in a sealed chamber at the 22th day after tumor implantation. The tibialis anterior, gastrocnemius muscles and tumors were harvested immediately after sacrificing mice and weighed. Part of the tibialis anterior and gastrocnemius muscle tissue samples were fixed in 4% paraformaldehyde and embedded in paraffin for subsequent studies. Other tissue samples were immediately frozen in liquid nitrogen and stored under − 80 °C.

### Myofiber cross-sectional area measurement

Hematoxylin and eosin (H&E) staining was performed on a middle cross-section of the tibialis anterior and gastrocnemius muscles to determine the myofiber cross-sectional area (CSA). The CSA of H&E stained muscle sections was determined using ImageJ software (National Institutes of Health, Bethesda, MD). A total of 200 myofibers within each section were measured in four different view fields. Measurements were performed blindly [24].

### Immunohistochemistry (IHC)

The tissue of skeletal muscle was fixed with 4% paraformaldehyde, dehydrated, embedded in paraffin, and sliced at 4 μm. After standard dewaxing and rehydration, EDTA (PH9.0, Bios Biological) was then used for antigen retrieval for 90 s. After antigen retrieval, activity of endogenous peroxidase was blocked with 3% H_2_O_2_. Sections were then blocked with fetal bovine serum and incubated with a primary antibody solution overnight at 4 °C. Then, after rewarming, the slides were incubated with secondary antibody at room temperature for 25 min. Last, the sections were rinsed in tap water. After drying, the sections were sealed with a neutral resin, covered the coverslip, dried naturally.

### Myogenic cell culture and differentiation

C2C12 myoblasts were cultured in Dulbecco’s modified Eagle medium (DMEM, Thermo Fisher, Waltham, MA, USA) supplemented with 10% fetal bovine serum (FBS) and 1% penicillin–streptomycin. Cells were then incubated at 37 °C in a 5% CO_2_ environment. After attaining 60–70% confluence, differentiation of cells was induced by incubation for four days in a differentiation medium: DMEM supplemented with 2% horse serum (Gibco, Gaithersburg, MD) [25].

### LLC cell culture and conditioned medium preparation

LLC cells were cultured in DMEM supplemented with 10% FBS and 1% penicillin–streptomycin, and incubated at 37 °C in a 5% CO_2_ environment. For conditioned medium collection, the medium was changed to DMEM when LLC cells reached a 95–100% confluence and were collected 48 h after media change. C2C12 myotubes were treated with LLC cell-conditioned medium (LCM) for 48 h after the differentiation protocol. LCM comprised 25% LLC-cell-conditioned medium and 75% DMEM.

### Diameter of C2C12 myotubes measurement

Brightfield microscopy was used to obtain C2C12 myotube images for further morphometric studies. Ten view fields (10 myotubes in each area) within each group were analyzed using ImageJ software.

### RNA isolation and real-time PCR analysis

Total RNA was extracted from cultured cells and frozen tissues using TRizol reagent (Thermo Fisher, Waltham, MA, USA) according to the manufacturer’s instructions. RNA purity and concentration were determined using a NanoDrop 2000 Spectrophotometer (NanoDrop Technologies, Wilmington, USA). Reverse transcription was performed using 1 μg of the extracted RNA. qRT-PCR was conducted with a SYBR Green master mix (YEASEN, China) in a 96-well format with StepOnePlus™ Real-Time PCR System (Thermo Fisher Scientific, USA). GAPDH was used to normalize the expression levels of the samples. U6 was used as housekeeping gene for miRNAs. All reactions were run in triplicates. Relative expression levels of miR-29c and LIF were calculated using 2-ΔCт method (ΔCт = Cт^gene^ − Cт^normalizer^). The following sets of primer pairs were used in this study: LIF (Forward: 5′-ATTGTGCCCTTACTGCTGCTG-3′, Reverse: 5′-GCCAGTTGATTCTTGATCTGGT-3′). GAPDH (Forward: 5′-GGTGAAGGTCGGAGTCAACGG-3′, Reverse: 5′-GAGGTCAATGAAGGGGTCATTG-3′). U6 (Forward: 5′-CTCGCTTCGGCAGCACA-3′, Reverse: 5′-AACGCTTCACGAATTTGCGT-3′).

### Western blotting

Protein extraction was performed using RIPA protein lysis buffer (Beyotime Institute of Biotechnology, Haimen, China) with a freshly added protease inhibitor cocktail and PMSF. The extracted protein (30 µg) was subjected to SDS-PAGE and transferred to a polyvinylidene fluoride (PVDF) membrane. The membrane was then blocked with 5% non-fat skimmed milk for 1 h at room temperature after which it was incubated overnight with a primary antibody. Membranes were washed with PBST to remove excess primary antibodies and then incubated for 2 h with secondary antibodies in a blocking buffer before detection. Enhanced chemiluminescence (ECL) western blotting substrates (Thermo Fisher, Waltham, MA, USA) were used for visualization of protein expression.

### Transfection of siRNA

C2C12 cells are seeded in 6-well plates. When the confluence reaches 60%, cells were transfected with siRNA (0.4 nmol) per well or its control using Lipofectamine 2000 reagent (Invitrogen, Carlsbad, CA, USA), following the manufacturer’s protocols. The medium was changed to DMEM supplemented with 10% FBS after 10 h. The myotubes were harvested after 48 h for further studies. The following siRNA were used in this study: siRNA-1: GAACCAGATCAAGAATCAA. siRNA-2: CAAGCTCAATGCTACTATA. siRNA-3: CGACCACTCTGACAAAGAA.

### Transfection of the miRNA mimic and inhibitor

LLC cells are seeded in 6-well plates. When the confluence reaches 70%, cells were transfected with miRNA mimic (0.2 nmol), inhibitor (0.8 nmol), and the control (miR-NC) into LLC cells using Lipofectamine 2000 reagent, following the manufacturer's protocols. LLC cells were transferred to a fresh medium after 10 h for further incubation to obtain the conditioned medium.

### Fluorescence microscopy analysis

C2C12 myotubes were stained using an anti-MHC antibody followed by Alexa Fluor^®^ 593- conjugated secondary antibody. Myotubes were then imaged using an OLYMPUS FV3000 microscope at × 40 magnification to determine myotube diameter.

### Luciferase reporter assay

Reporter plasmids were constructed by Guangzhou RiboBio (RiboBio, Guangzhou, China). 293T cells were seeded in 24-well plates. After reaching a confluence of 30%, cells were transfected with 100 ng of LIF-WT or LIF-MUT, along with 50 nM of miR-29c mimics using Lipofectamine 2000 (Invitrogen, Carlsbad, CA, USA) according to the manufacturer's instructions. Cells were harvested 24 h after transfection and lysed for luciferase assays. Luciferase activity was determined using the dual-luciferase reporter reagent (Promega, E1910, Madison, WI, USA) according to the manufacturer's instructions. Each sample was assayed in triplicate.

### Statistical analysis

In vitro experiments were performed in triplicates to ensure high statistical power. Statistical differences between and among groups were evaluated using Student’s t-test and ANOVA followed by Tukey’s post hoc tests, respectively. GraphPad Prism 8 software was used for statistical analyses and *P* < 0.05 was set as the threshold for statistical significance.

## Results

### Low expression levels of miR-29c in lung adenocarcinoma (LUAD) tissues is correlated with survivals

**Fig. 1:**
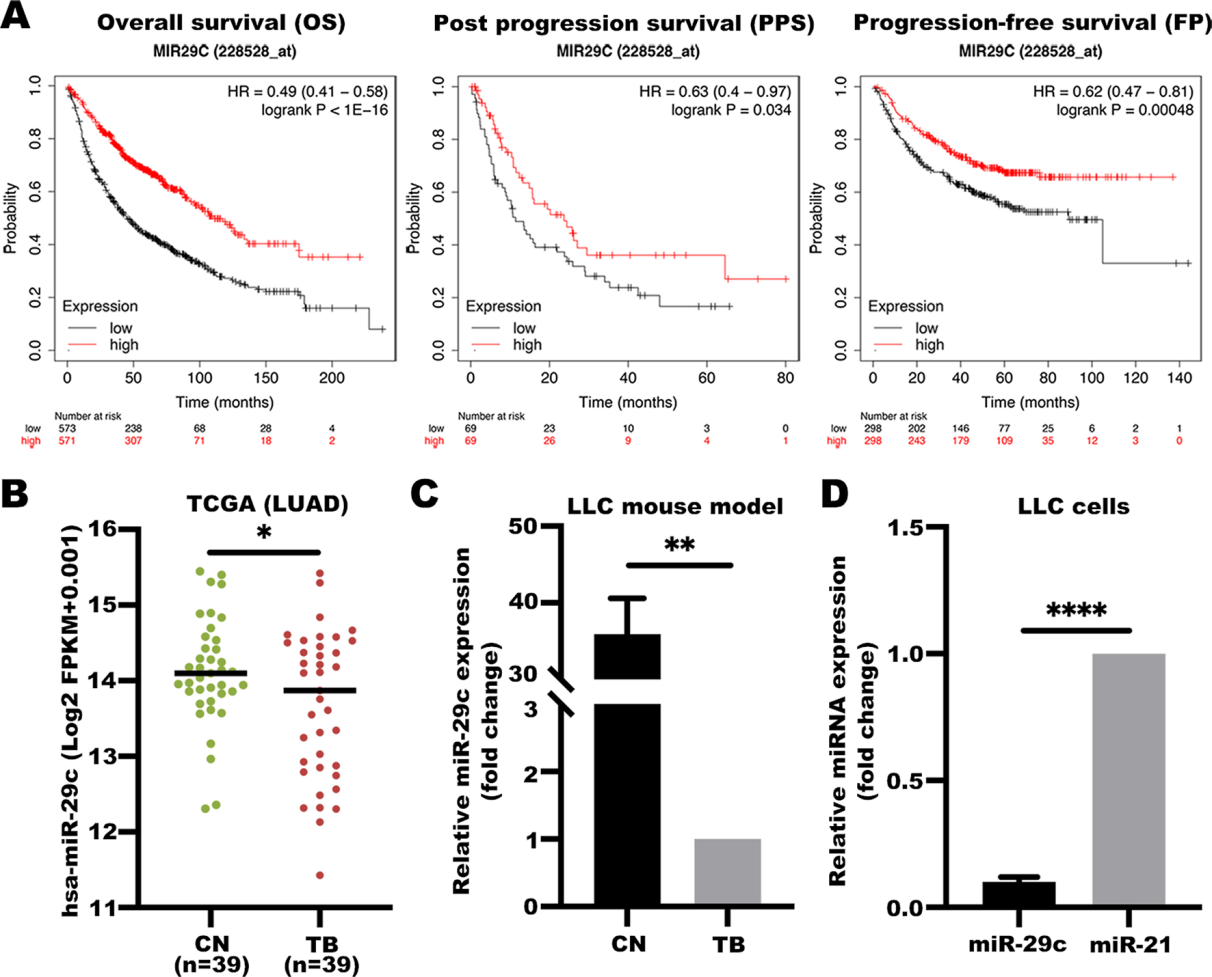
Low expression levels of miR-29c in lung adenocarcinoma (LUAD) tissues is correlated with survival **A** The Kaplan–Meier curves of miR-29c in LUAD for overall survival (OS), post-progression survival (PPS), and progression-free survival (FP). **B** miR-29c is downregulated in LUAD. Expression levels of miR-29c in 39 LUAD tissues and paired normal tissues based on data from the TCGA database. Student’s *t*-test, *P < 0.05. **C** miR-29c is repressed in LLC mouse model. The expression levels of miR-29c in tumors of LLC tumor-bearing mice and lungs in control group mice were examined by using qRT-PCR analyses. Data (*n* = 3) were analyzed by Student *t*-test. **P < 0.01. **D** miR-29c is downregulated in LLC cells. The expression levels of miR-29c and miR-21 in LLC cells were examined by using qRT-PCR analyses. Data (*n* = 3) were analyzed by Student’s *t*-test, ****P < 0.0001

Previous studies reported that miR-29 family plays a role as a tumor suppressor in several cancer types. Downregulation of miR-29 expression leads to deviations in various cellular biological functions [26]. Also, studies have shown miR-29c is involved in myotube synthesis and differentiation [22]. Although effects of downregulation of miR-29c in different cancer types have been explored, their functions in lung cancer cachexia have not been reported. To elucidate the biological functions of miR-29c in muscle atrophy in lung cancer cachexia, LUAD data were retrieved from public databases and Kaplan Meier Plotter tools (https://kmplot.com/analysis/) were used for survival analysis. Bioinformatic analysis showed that high miR-29c expression level in LUAD patients was significantly correlated with improved overall survival (OS), post-progression survival (PPS), and progression-free survival (FP) outcomes [27] (Fig. 1A).

To validate the bioinformatic findings, data of 39 LUAD cases and paired normal cases were downloaded from TCGA database and analyzed. Paired normal cases were derived from normal lung tissues or adjacent tissues of the corresponding LUAD patients for use as controls. The findings showed that miR-29c was significantly downregulated in LUAD compared with the control (Fig. 1B). Furthermore, we used C57BL/6 mouse to build the Lewis lung carcinoma-induced cachexia model (LLC model), and tried to compared the miR-29c levels between tumors in TB mice and normal lungs in CN mice. Figure 1C shows that miR-29c was repressed in tumors compared with control. miR-21 is upregulated in several types of cancer, including lung cancer. Studies have shown its role as an oncogene and increased expression of miR-21 associated with a worse outcome within lung cancer patients [28–30]. Therefore, we chose miR-21 as a positive control in LLC cells. Figure 1D shows that miR-29c is downregulated compared to miR-21. Therefore, miR-29c was selected as a target molecule for subsequent studies.

### Downregulation of miR-29c expression induced muscle wasting in cachexia

The findings showed that miR-29c levels were significantly downregulated in lung cancer, therefore, miR-29c may be implicated in the occurrence and development of lung cancer cachexia. To explore the correlation between miR-29c expression levels and cachectic phenotype, C2C12 myotubes were treated with a conditioned medium of LLC cells (LCM) transfected with a miR-29c mimic or inhibitor referred as the cachexia model. Levels of atrophy-associated genes (atrogin1 and MuRF1), and autophagy markers (P62 and LC3-II), were then determined.

**Fig. 2:**
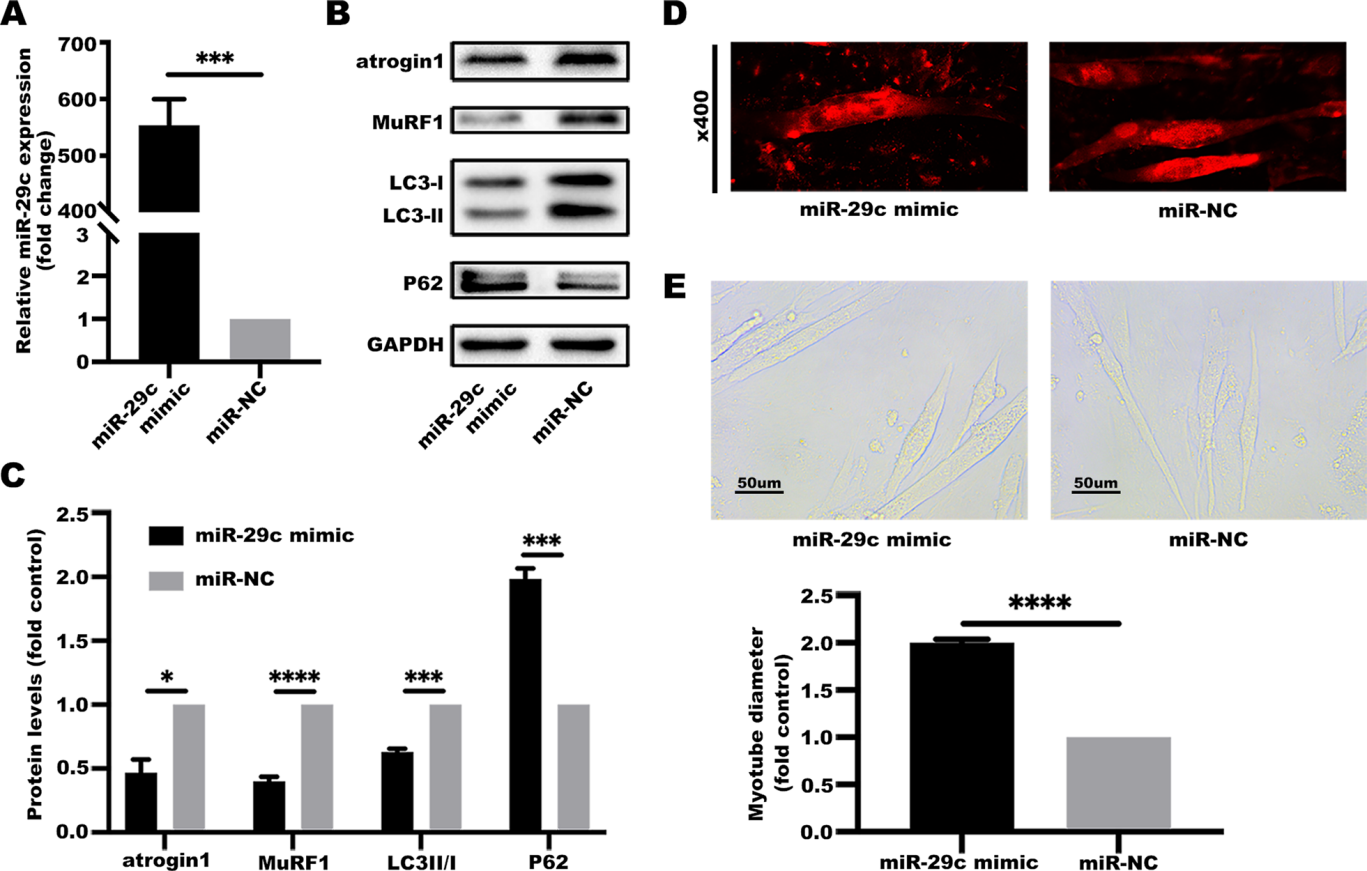
High expression of miR-29c in LLC cells ameliorate muscle catabolism and myotube atrophy. **A** The expression level of miR-29c is elevated in LLC cells after miR-29c mimic transfection. microRNA level of miR-29c was determined by qRT-PCR analyses. Data (n = 3) were analyzed by Student’s *t*-test, ***P < 0.001. **B**, **C** High expression of miR-29c in LLC cells ameliorate catabolic response in C2C12 myotubes. C2C12 myotubes were treated with a conditioned medium of LLC cells transfected with a miR-29c mimic or its negative control for 48 h. The protein involved in catabolic response were evaluated by western blotting. Data (n = 3) were analyzed by Student’s *t*-test, *P < 0.05, ***P < 0.001, ****P < 0.0001. **D** High expression of miR-29c in LLC ameliorate C2C12 myotube atrophy. C2C12 myotubes were treated as described in **B**, **C** and analyzed for myotube diameter of MHC-stained myotubes (at 48 h). **E** High expression of miR-29c in LLC ameliorate C2C12 myotube atrophy. C2C12 myotubes were treated as described in **B**, **C** and analyzed for myotube diameter (at 48 h). Data (n = 3) were analyzed by Student’s *t*-test, ****P < 0.0001

miR-29c was overexpressed in LLC cells (Fig. 2A). In the miR-29c overexpression group, upregulation of atrogin1, MuRF1, and LC3-II was partially inhibited while P62 loss was relatively prevented in the C2C12 myotubes (Fig. 2B, C). Analysis of myotube diameter showed that myotube atrophy had been ameliorated (Fig. 2D, E).

**Fig. 3:**
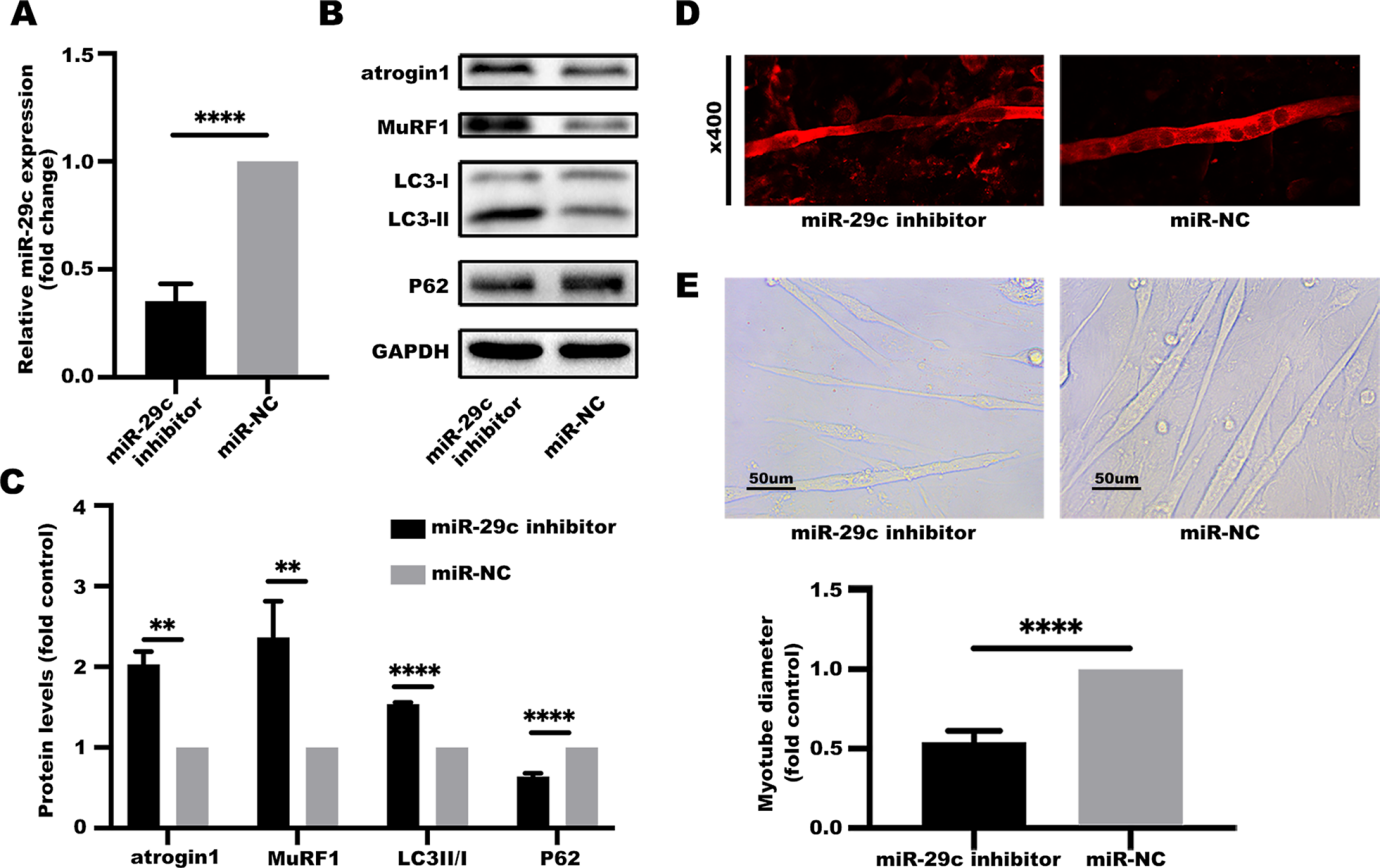
Suppressed expression of miR-29c in LLC cells induce muscle catabolism and myotube atrophy. **A** The expression level of miR-29c is repressed in LLC cells after miR-29c inhibitor transfection. microRNA level of miR-29c was determined by qRT-PCR analyses. Data (n = 3) were analyzed by Student's *t*-test, ****P < 0.0001. **B**, **C** Suppressed expression of miR-29c in LLC cells induces a catabolic response in C2C12 myotubes. C2C12 myotubes were treated with a conditioned medium of LLC cells transfected with a miR-29c inhibitor or its negative control for 48 h. The protein involved in catabolic response were evaluated by western blotting. Data (n = 3) were analyzed by Student’s *t*-test, *P < 0.05, **P < 0.01, ****P < 0.0001. **D** Suppressed expression of miR-29c in LLC induce C2C12 myotube atrophy. C2C12 myotubes were treated as described in **B**, **C** and analyzed for myotube diameter of MHC-stained myotubes (at 48 h). **E** Suppressed expression of miR-29c in LLC induce C2C12 myotube atrophy. C2C12 myotubes were treated as described in **B**, **C** and analyzed for myotube diameter (at 48 h). Data (n = 3) were analyzed by Student's *t*-test, ****P < 0.0001

LLC cells were transfected with miR-29c inhibitor to further downregulate miR-29c expression (Fig. 3A). The findings showed that repression of miR-29c induced muscle catabolism (Fig. 3B, C) and wasting (Fig. 3D, E). These findings imply that miR-29c plays a crucial role in pathogenesis of muscle wasting caused by lung cancer-induced cachexia. Therefore, miR-29c is a potential target for treatment of skeletal muscle deficiency in cancer cachexia.

### miR-29c exerts its activity by targeting LIF, a cachexia-inducing factor

**Fig. 4:**
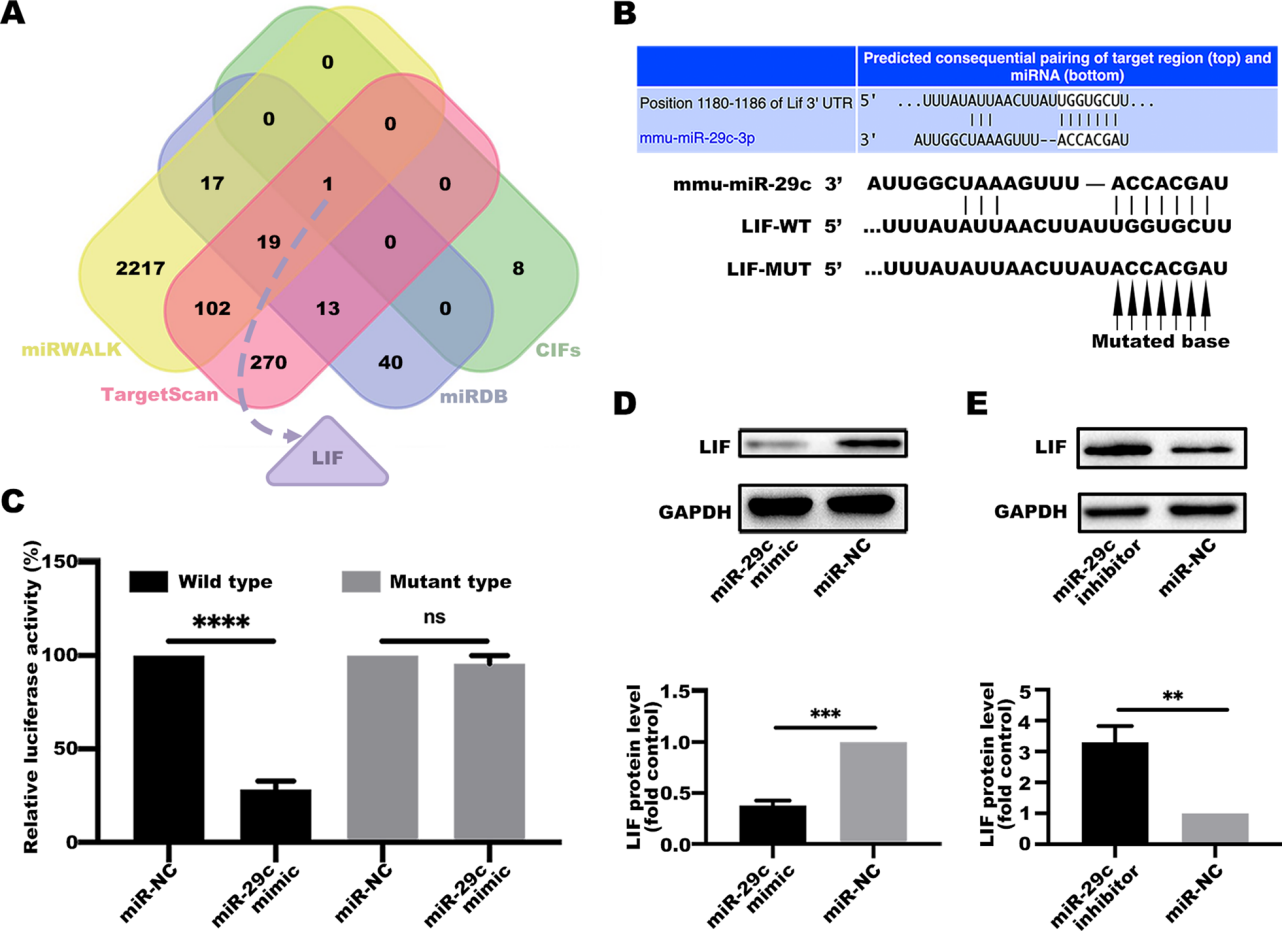
miR-29c exerts its activity by targeting LIF, a cachexia-inducing factor. **A** Venn diagram for potential target gene sets of miR-29c from miRWALK (http://mirwalk.umm.uni-heidelberg.de/), TargetScan (http://www.targetscan.org/) miRDB (http://mirdb.org/) and datasets of high expression of cachexia-inducing factors. **B** Construction of luciferase plasmids containing LIF 3′-UTR or LIF 3′-UTR mutant. **C** LIF is a potential target gene of miR-29c. Determination of luciferase activity in 293T cells. Data (n = 3) were analyzed by Student’s *t*-test, ****P < 0.0001, ns *P* > 0.05. **D** C2C12 myotubes were treated with a conditioned medium of LLC cells transfected with a miR-29c mimic or its negative control for 48 h. The protein of LIF was evaluated by western blotting. Data (n = 3) were analyzed by Student’s *t*-test, ***P < 0.001. **E** C2C12 myotubes were treated with a conditioned medium of LLC cells transfected with a miR-29c inhibitor or its negative control for 48 h. The protein of LIF was evaluated by western blotting. Data (n = 3) were analyzed by Student’s *t*-test, ***P < 0.001

miRWALK, TargetScan and miRDB tools and datasets of high expression of cachexia-inducing factors (CIFs) were used to explore the mechanisms through which miR-29c overexpression alleviates the cachectic phenotype [31]. The leukemia inhibitory factor (LIF) gene was identified as a promising miR-29c target in lung cachexia (Fig. 4A). To validate the direct interaction between miR-29c and LIF, the miR-29c binding site at the 3′ UTR of LIF mRNA (LIFwt-3'UTR) or its mutant (LIFmut-3′ UTR) was cloned downstream of the firefly luciferase reporter gene. The clone was then co-transfected with the miR-29c mimic or its negative control into 293T cells (Fig. 4B). The construction sequences are presented in Additional file 1: Fig. S1. The relative luciferase activity of the reporter containing LIFwt-3′ UTR was significantly decreased after co-transfection of the miR-29c mimic compared with the activity of the reporter containing the LIFmut-3′ UTR (Fig. 4C).

Further, miR-29c was overexpressed in LLC cells and cultured with C2C12 myotubes in conditional media and then LIF protein levels were determined. LIF protein level was downregulated in the miR-29c overexpressed group (Fig. 4D), whereas LIF protein level upregulated in the miR-29c inhibited group (Fig. 4E). These findings indicate that miR-29c modulates LIF expression by directly binding to its 3'UTR.

### LIF promotes cancer-induced muscle wasting through JAK/STAT and MAP-kinase pathways

**Fig. 5:**
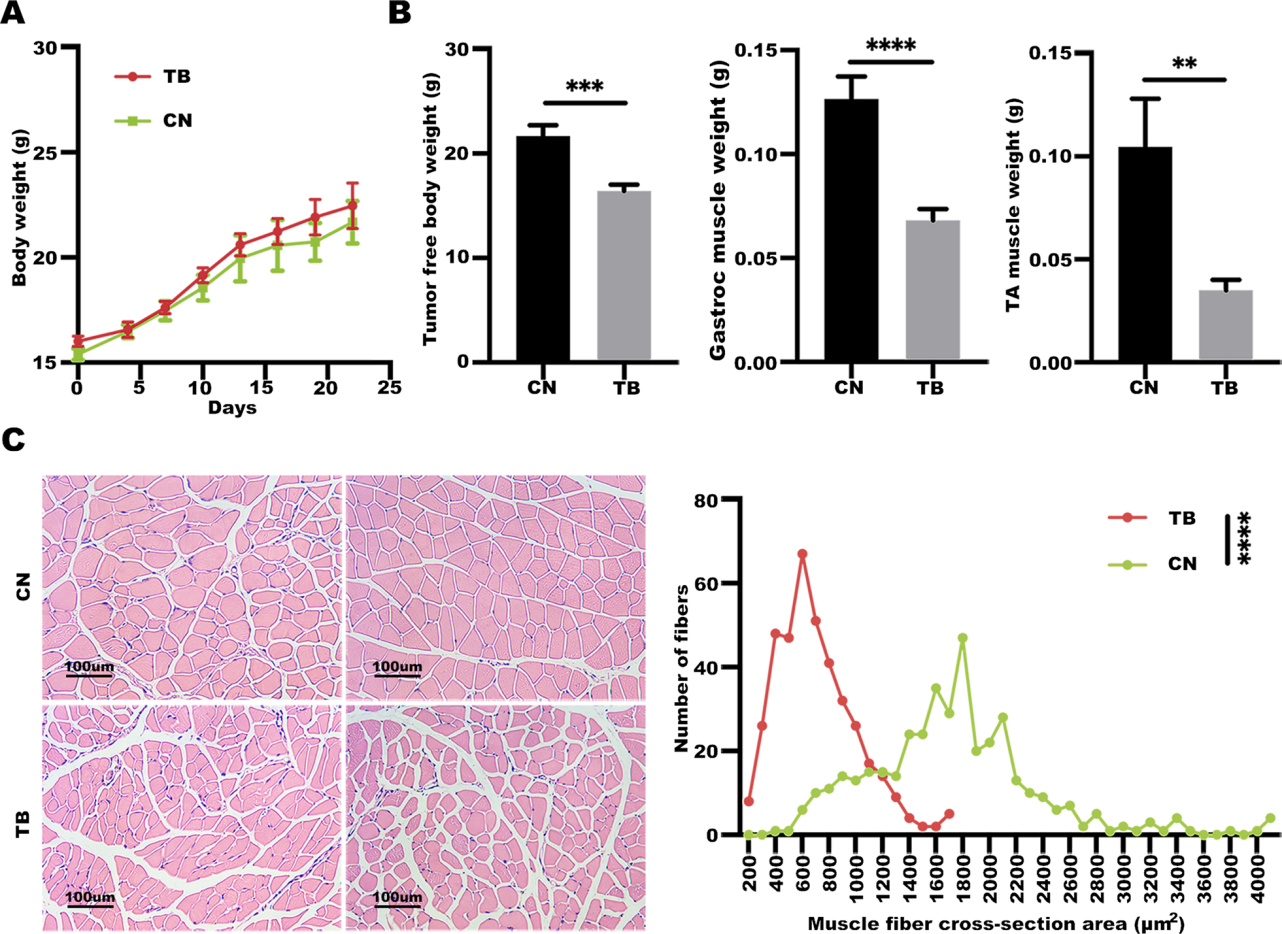
LLC tumor-induced cancer cachexia cause weight loss and skeletal muscle weakness. **A** The body weight growth curves of C57BL/6 mice in 22 days after LLC cells and PBS injection. **B** LLC tumor-bearing mice suffer from cancer cachexia. Weight changes of the tumor-free body, gastrocnemius and tibial anterior muscle between CN and TB group. Data (n = 8) were analyzed by *t*-test, **P < 0.01, ***P < 0.001, ****P < 0.0001. **C** LLC tumor-bearing mice suffer from muscle wasting. Representative images of H&E stained middle cross-section of the gastrocnemius from CN and TB mice. Data (n = 8) were analyzed by χ^2^ analysis, ****P < 0.0001

High miR-29c levels can ameliorate the lung cachectic phenotype and directly regulate LIF protein expression, therefore, the current study hypothesized that LIF expression is correlated with cachexia. To test this hypothesis, Lewis lung carcinoma-induced cancer cachectic model (LLC model) was established using C57BL/6 mouse. The finding showed that the body weights of mice in the TB group were higher than those of the CN group, however, the differences were not significant (Fig. 5A). Notably, tumor-free body weights of mice in the TB group were significantly lower compared with the body weights of the control group mice. This change was not due to reduced food and water intake (Fig. 5B). Gastrocnemius and tibial anterior muscle weights were lower in the TB group compared with those in the control group. Hematoxylin and eosin staining revealed that muscle fiber cross-sectional area (CSA) of the gastrocnemius and tibialis anterior were significantly less in mice in the TB group compared with those of mice in the CN group (Fig. 5C).

**Fig. 6:**
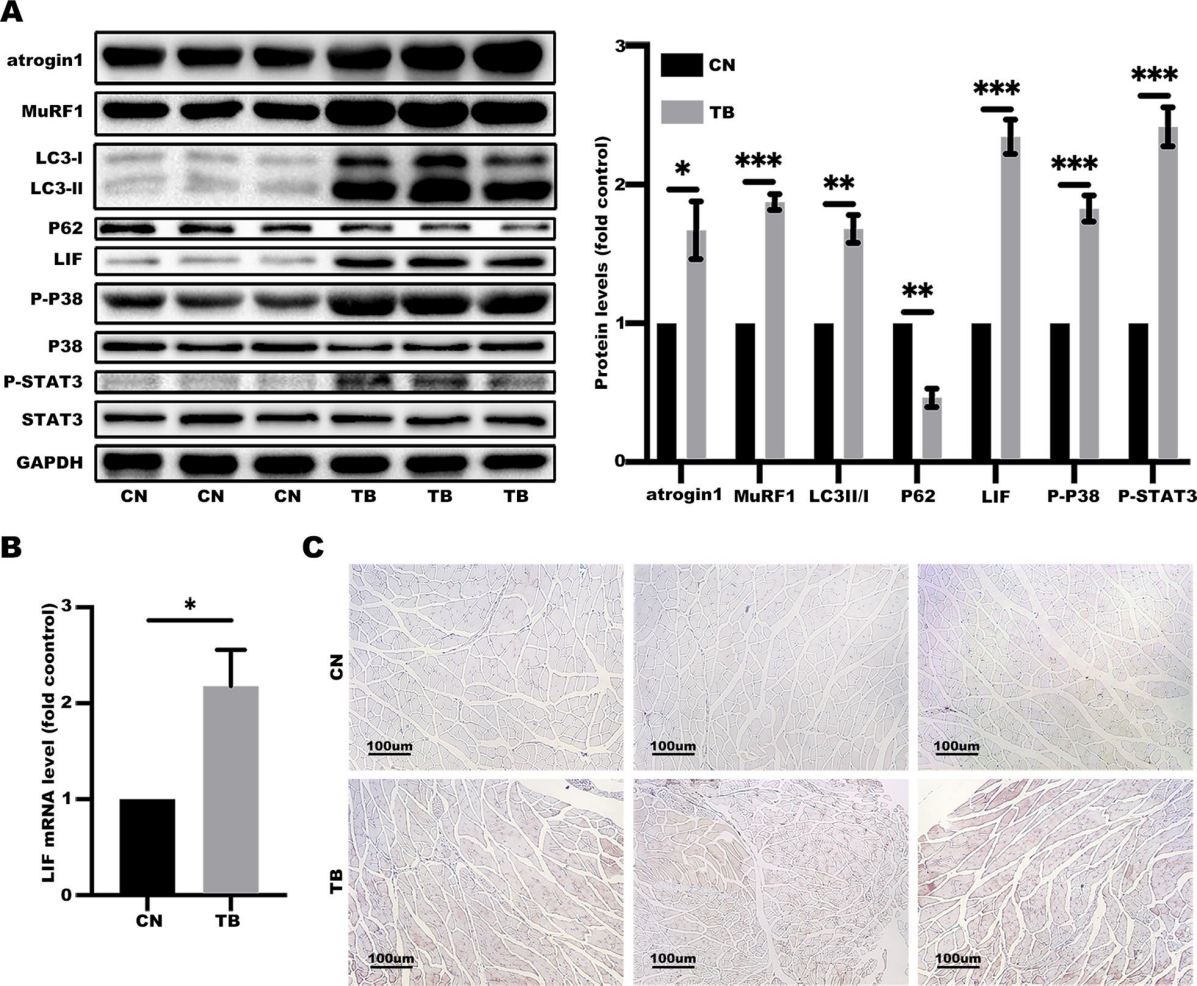
LIF and related signaling pathways are activated in LLC cachectic model. **A** The protein involved in catabolic response and LIF signaling pathways in gastrocnemius tissues were evaluated by western blotting. Data (n = 3) were analyzed by Student’s *t*-test, *P < 0.05; **P < 0.01; ***P < 0.001. **B** LIF is elevated in the skeletal muscle of cachexia mice. The mRNA level of LIF was determined by qRT-PCR analyses. Data (n = 3) were analyzed by Student’s *t*-test, *P < 0.05. **C** Representative immunohistochemistry (IHC) staining for LIF in gastrocnemius tissues (Brown represents the protein of LIF)

**Fig. 7:**
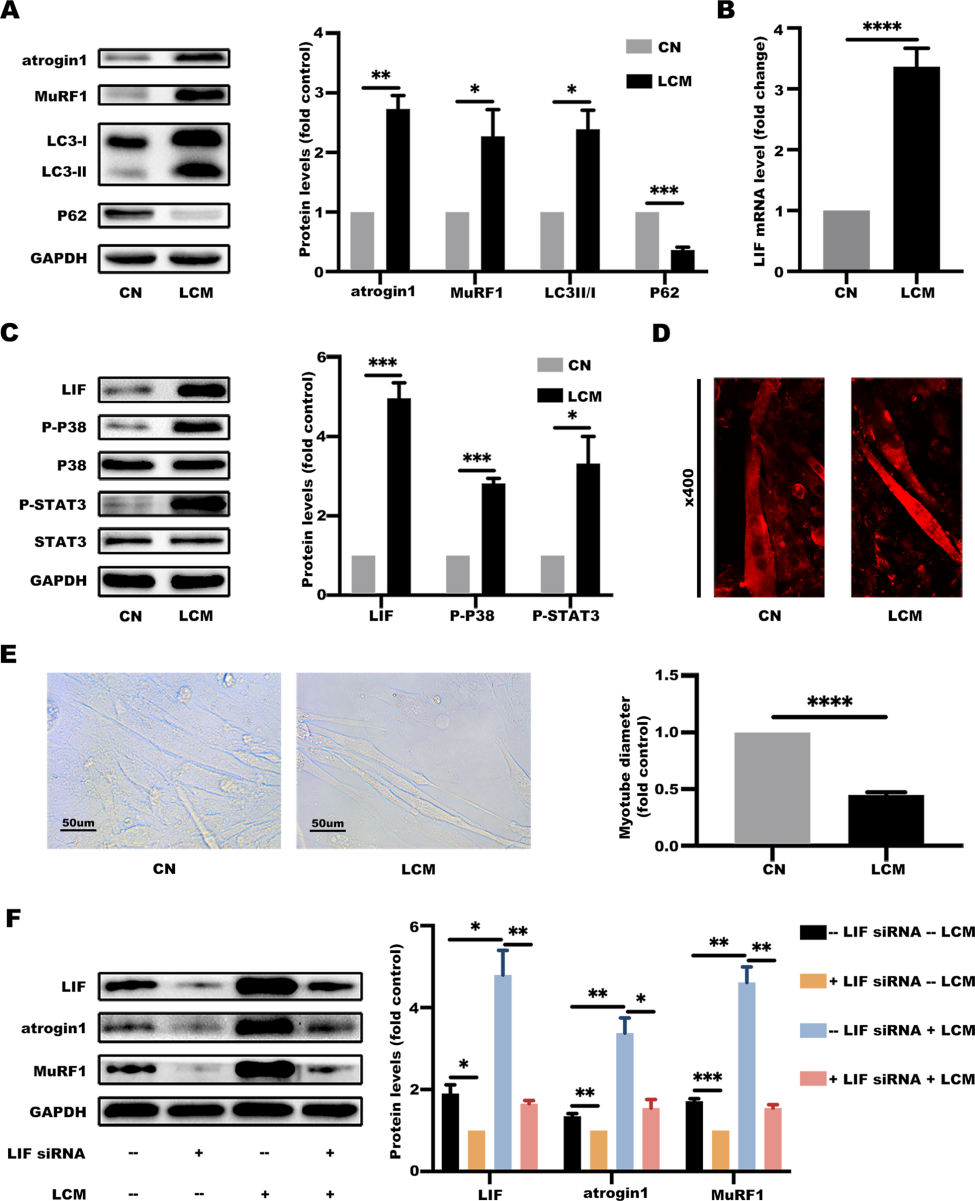
LIF promotes cancer-induced muscle wasting through the JAK/STAT and MAP-kinase pathways. **A** LCM induces catabolic response in C2C12 myotubes. C2C12 myotubes were treated with a conditioned medium of LLC cells for 48 h. The protein involved in catabolic response were evaluated by western blotting. Data (n = 3) were analyzed by Student’s *t*-test, *P < 0.05; **P < 0.01; ***P < 0.001. **B** LIF is elevated in C2C12 myotubes. C2C12 myotubes were treated as described in **A**. The mRNA level of LIF in C2C12 myotubes was determined by using qRT-PCR analyses. Data (n = 3) were analyzed by Student's *t*-test, ****P < 0.0001. **C** LIF and its signaling pathway is activated in C2C12 myotubes. C2C12 myotubes were treated as described in **A**. The protein involved in LIF signaling pathways were evaluated by western blotting. Data (n = 3) were analyzed by Student’s *t*-test, *P < 0.05; ***P < 0.001. **D** LCM induces C2C12 myotube atrophy. C2C12 myotubes were treated as described in **A**. Representative immunofluorescence (MHC, red) images of C2C12 myotubes. **E** LCM induces C2C12 myotube atrophy. C2C12 myotubes were treated as described in **A** and analyzed for myotube diameter (at 48 h). Data (n = 3) were analyzed by Student’s *t*-test, ****P < 0.0001. **F** miR-29c directly targets LIF. C2C12 myotubes were transfected with LIF siRNA and treated with a conditioned medium of LLC cells for 48 h. The protein levels of LIF, atrogin1, and MuRF1 in C2C12 myotubes were evaluated by western blotting. Data (n = 3) were analyzed by Student's *t*-test, *P < 0.05; **P < 0.01; ***P < 0.001

Studies report that LIF signaling pathways include JAK/STAT and MAP-kinase pathways [32]. To explore whether these two signaling pathways are involved in pathogenesis of the lung cancer cachexia, levels of atrophy-associated genes, autophagy markers, LIF expression, and protein expression levels of p-p38 and p-STAT3 in gastrocnemius were determined (Fig. 6A–C). Protein and mRNA expression levels of LIF were significantly higher, and JAK/STAT and p38 MAPK pathways in the cachectic model were highly activated compared with the control group. Similar findings were obtained in analysis using cell lines. Treatment of C2C12 myotubes with LCM increased expression levels of atrophy-associated genes, activated autophagy markers, LIF and related signaling pathways (Fig. 7A–C). Notably, miR-29c expression levels in the gastrocnemius and C2C12 myotubes were downregulated by LCM treatment (Additional file 1: Figs. S2, S3). Moreover, the diameter of myotubes in the LCM group was significantly thinner compared with the control group (Fig. 7D, E).

Three independent siRNAs were designed targeting LIF. The most effective one (siRNA-3) was selected for subsequent studies to validate the molecular mechanism of LIF and atrophy-associated genes (Additional file 1: Fig. S4). LIF siRNA or its negative control was transfected into C2C12 myotubes, which were treated with LCM. Downregulation of LIF levels promoted reduction of atrogin1 and MuRF1 levels (Fig. 7F). These findings support the hypothesis that LIF promotes cancer-induced muscle wasting through JAK/STAT and MAP-kinase pathways.

### Downregulation of miR-29c induces muscle wasting by modulating LIF activity

**Fig. 8:**
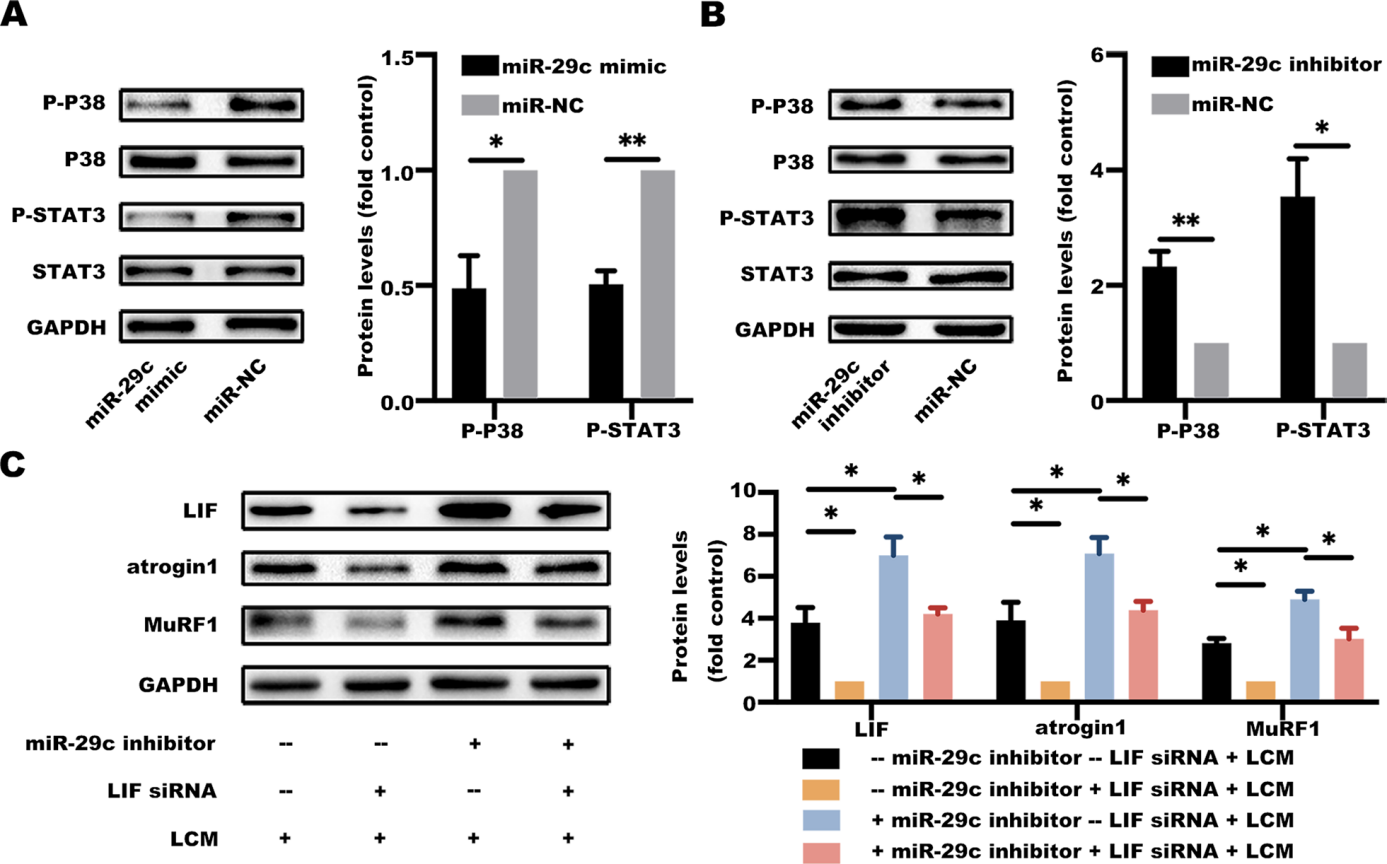
Downregulation of miR-29c induces muscle wasting by modulating LIF activity. **A** High expression of miR-29c in LLC inhibit LIF signaling pathways. C2C12 myotubes were treated with a conditioned medium of LLC cells transfected with a miR-29c mimic or its negative control for 48 h. The protein involved in LIF signaling pathways were evaluated by western blotting. Data (n = 3) were analyzed by Student’s *t*-test, *P < 0.05; **P < 0.01. **B** Suppressed expression of miR-29c in LLC activate LIF signaling pathways. C2C12 myotubes were treated with a conditioned medium of LLC cells transfected with a miR-29c inhibitor or its negative control for 48 h. The protein involved in LIF signaling pathways were evaluated by western blotting. Data (n = 3) were analyzed by Student’s *t*-test, *P < 0.05; **P < 0.01. **C** miR-29c directly targets LIF. C2C12 myotubes were transfected with LIF siRNA and treated with a conditioned medium of LLC cells transfected with a miR-29c inhibitor for 48 h. The protein levels of LIF, atrogin1, and MuRF1 were evaluated by western blotting. Data (n = 3) were analyzed by Student’s *t*-test, *P < 0.05

The mechanism of muscle wasting mediated by LIF due to decrease of miR-29c was further explored. Protein expression levels of p-p38 and p-STAT3 in C2C12 myotubes treated with conditioned medium of LLC cells transfected with miR-29c mimic or inhibitor were determined. JAK/STAT and MAP-kinase pathways were inhibited in the group with highly expressed miR-29c, whereas the two pathways were activated in the miR-29c downregulated group (Fig. 8A, B).

A functional response experiment was performed by transfecting LIF siRNA in C2C12 myotubes. The C2C12 myotubes were then treated with a conditioned medium of LLC cells transfected with a miR-29c inhibitor. Western blotting assays showed that miR-29c knockdown increased atrogin1 and MuRF1 expression levels, whereas LIF siRNA inhibited atrogin1 and MuRF1 expression. In addition, miR-29c knockdown-induced overexpression of atrogin1 and MuRF1 was reversed by LIF inhibition (Fig. 8C). These findings show that miR-29c induces muscle wasting through modulation of LIF activity.

## Discussion

miR-29c was downregulated in cachexia-inducing lung cancer, and its expression was negatively correlated with muscle catabolism. Notably, inhibition of miR-29c expression stimulated muscle catabolism by activating LIF on muscle myotubes. The findings of the current study show that LIF promotes cancer-induced muscle wasting by modulating JAK/STAT and MAP-kinase pathways. These findings indicate that miR-29c enhances muscle wasting by upregulating LIF levels in muscles.

MicroRNAs are a group of endogenous single-stranded small (19 ~ 23 nucleotides) noncoding RNA molecules. MicroRNAs act as oncogenes or tumor suppressors, thus they are implicated in cancer initiation and development [33, 34]. Several overexpressed miRNAs act as oncogenes by targeting important tumor suppressors. For example, overexpressed miR-21 promotes hepatocellular carcinoma progression and metastasis by targeting PTEN expression [35]. Several miRNAs that function as tumor suppressors have been reported. Croce et al. reported that loss of function of miR-15a and miR-16-1 leads to chronic lymphocytic leukemia [36]. Moreover, miR-29 s expression is negatively correlated with DNMT3A and -3B in lung cancer [37]. The miR-29 family comprises three members, including miR-29a, miR-29b, and miR-29c, and all members function as potent tumor suppressor genes in various cancers [26]. Previous studies report that miR-29a regulates tumor growth and migration by targeting CLDN1 in hepatocellular carcinoma [38]. The findings in this study showed that miR-29c expression was downregulated in LUAD and LLC cells compared with normal cells. Moreover, loss and gain of function in vitro assays showed that muscle catabolic activity was associated with low levels of miR-29c in the cachexia model. Therefore, miR-29c was correlated with cachexia and muscle wasting, implying that it is a potential therapeutic target for cancer cachexia.

LIF is a multi-functional member of the interleukin-6 family of cytokines, and plays an intricate role in cancer [32]. Although LIF acts as a tumor suppressor in leukemia, studies report that LIF is overexpressed in different cancer types and is correlated with poor prognosis [39, 40]. LIF is a negative modulator of p53 in colorectal cancer [41]. In addition, LIF acts as CIFs in LUAD and is enriched in patients with low survival outcomes [31]. Previous studies report that the interaction between LIF and its receptor promotes activation of JAK1. Subsequently, JAK1 stimulates three individual signaling pathways, including JAK/STAT, MAP-kinase, and PI(3) Kinase pathways. Most effects of LIF on skeletal muscles are characterized as pleiotropic. LIF stimulates skeletal myoblast proliferation but inhibits myoblast differentiation into myotubes through ERK signaling pathways [42, 43]. Moreover, in vivo administration of LIF promotes skeletal muscle regeneration after injury [44]. However, only a few studies have explored the role of LIF on skeletal muscles in a cachectic environment. The current study established in vitro and in vivo LLC cachexia models and the findings showed higher LIF protein and mRNA expression levels in gastrocnemius and C2C12 of LLC group compared with control. Therefore, it was hypothesized that LIF induces muscle wasting by mainly activating the JAK/STAT and MAP-kinase pathways. These findings show that LIF plays an important role in mediating muscle catabolic activity in lung cancer cachexia.

miRNAs are effective non-invasive circulating biomarkers for early diagnosis of cachexia owing to the high stability [45]. Fabbri et al. reported that miR-21 and miR-29a from lung cancer cells target Toll-like receptor family (human TLR8 and murine TLR7) in macrophages to trigger a pro-metastatic inflammatory response. Therefore, circulating miR-21 and miR-29a in lung cancer are potential markers for cachexia monitoring and diagnosis [46]. Furthermore, miRNA-130a has a potential clinical value in prediction of cachexia in head and neck cancers. Patients with downregulated miR-130a levels are at a higher risk of developing cachexia compared with patients with elevated miR-130a levels [47]. The findings in this study showed that elevated miR-29c expression level in lung cancer was significantly associated with improved survival outcomes (Fig. 1A). Moreover, down-regulation of miR-29c promotes muscle wasting through LIF. Therefore, miR-29c is a potential novel biomarker for diagnosis of cachexia.

## Conclusions

In summary, downregulation of miR-29c expression promotes muscle catabolism in lung cancer by directly targeting LIF. The findings showed that LIF is a potential target for miR-29c in lung cancer, and therefore, low plasma levels of miR-29c can be a novel biomarker for cancer cachexia. Moreover, targeting miR-29c or LIF might provide new insight into developing potential therapeutic strategies for cancer cachexia.

## Supplementary Information

**Additional file 1.** **Figure S1.** The explicit construction sequences of LIFwt-3'UTR and LIFmut-3'UTR. **Figure S2.** miR-29c is downregulated in the skeletal muscle of cachexia mice. **Figure S3.** miR-29c is downregulated in the cachectic C2C12 myotubes. **Figure S4.** The protein levels of LIF upon transfection of 3 independent siRNAs. **Figure S5.** Hematoxylin-eosin (H&E) staining of the LLC tumors.

## Data Availability

Not applicable.

## Abbreviations

LUAD: Lung adenocarcinoma
LLC: Lewis lung carcinoma
LCM: LLC cell-conditioned medium
CSA: Cross-sectional area
CIFs: Cachexia-inducing factors
LIF: Leukemia inhibitory factor
UPP: Ubiquitin–proteasome pathway
ALP: Autophagy–lysosomal pathway
atrogin1: Muscle atrophy F-box protein
MuRF1: Muscle RING finger containing protein1
