# Supplementary material for: Downregulation of miR-29c promotes muscle wasting by modulating the activity of leukemia inhibitory factor in lung cancer cachexia
Source: Cancer Cell Int. 2021 Nov 27;21:627. doi: 10.1186/s12935-021-02332-w (PMC8626920; doi:10.1186/s12935-021-02332-w)
Supplement: Supplementary file 1 — Additional file 1. Figure S1. The explicit construction sequences of LIFwt-3'UTR and LIFmut-3'UTR. Figure S2. miR-29c is downregulated in the skeletal muscle of cachexia mice. Figure S3. miR-29c is downregulated in the cachectic C2C12 myotubes. Figure S4. The protein levels of LIF upon transfection of 3 independent siRNAs. Figure S5. Hematoxylin-eosin (H&E) staining of the LLC tumors. [file 12935_2021_2332_MOESM1_ESM.docx]

**
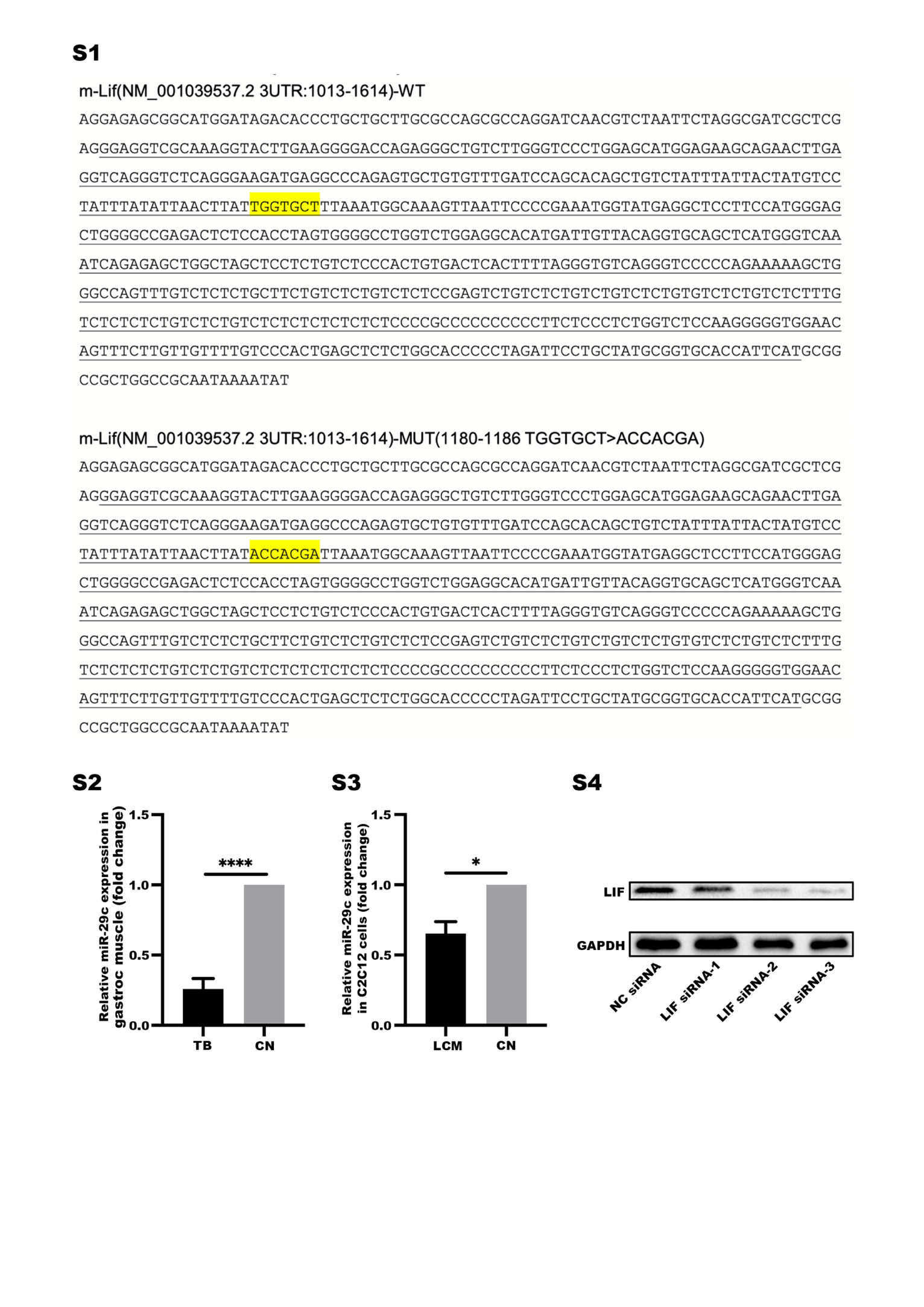
**

**
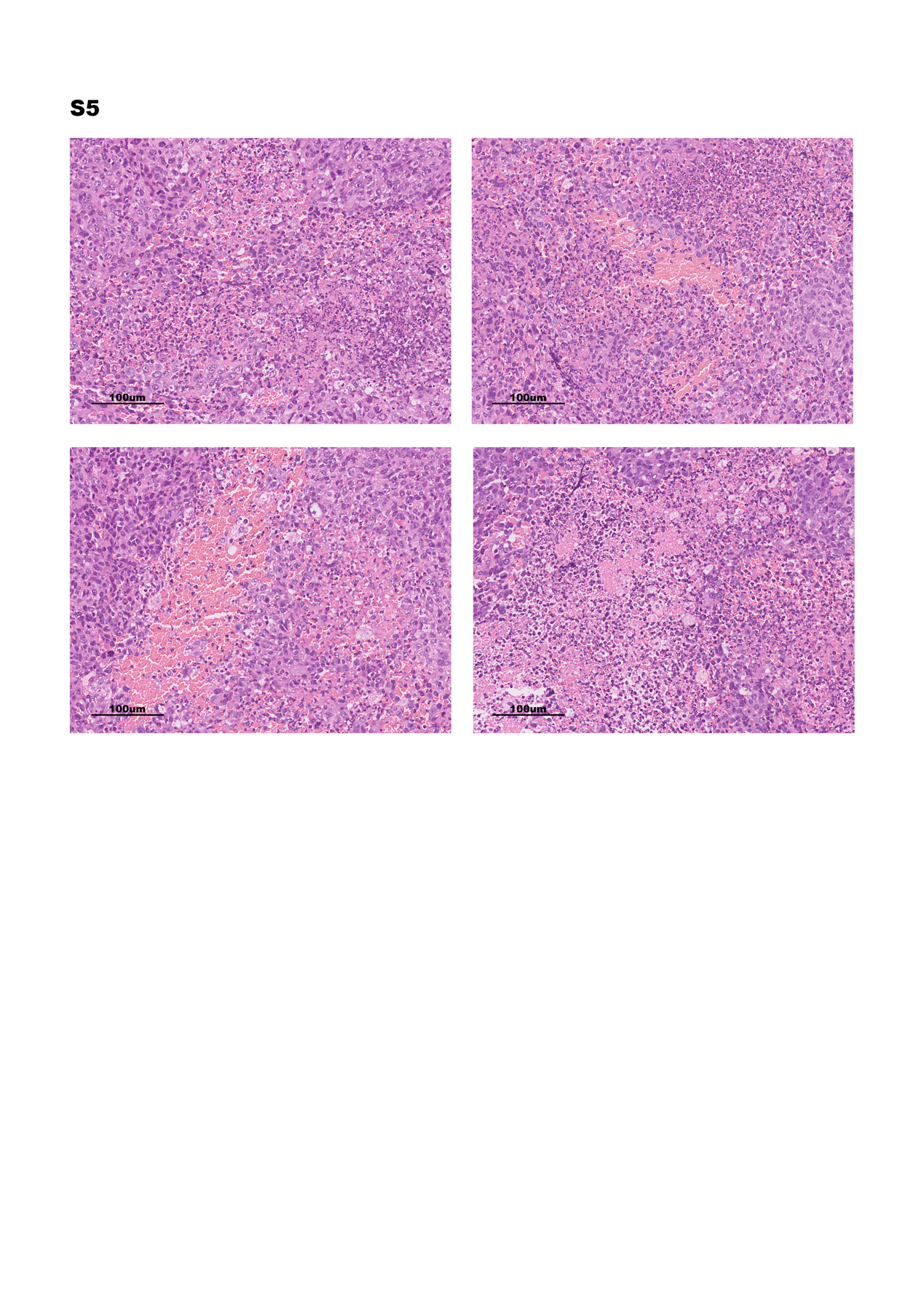
**

**Supplementary Figure** (S1) The explicit construction sequences of LIFwt-3'UTR and LIFmut-3'UTR. (S2) miR-29c is downregulated in the skeletal muscle of cachexia mice. microRNA level of miR-29c was determined by qRT-PCR analyses. Data (n=3) were analyzed by Student's *t*-test, *****P* < 0.0001. (S3) miR-29c is downregulated in the C2C12 myotubes. microRNA level of miR-29c was determined by qRT-PCR analyses. Data (n=3) were analyzed by Student's *t*-test, **P* < 0.05. (S4) The protein level of LIF upon transfection of 3 independent siRNAs was determined by qRT-PCR analyses. (S5) Hematoxylin-eosin (H&E) staining of the LLC tumours.
